# Narrowing but persisting gender pay gap among employees of the US Department of Health and Human Services during 2010–2018

**DOI:** 10.1186/s12960-021-00608-w

**Published:** 2021-05-13

**Authors:** Zhuo Chen, Yihong Zhang, Huabin Luo, Donglan Zhang, Janani Rajbhandari-Thapa, Yuheng Wang, Ruoxi Wang, Grace Bagwell-Adams

**Affiliations:** 1 Department of Health Policy and Management, College of Public Health, University of Georgia, 100 Foster Rd, 305B Wright Hall, Athens, GA 30602 United States of America; 2 School of Economics, Faculty of Humanities and Social Science, University of Nottingham Ningbo China, Ningbo, China; 3 Department of Public Health, East Carolina University, Greenville, NC United States of America; 4 Charles River Associates, Boston, MA United States of America; 5 School of Medicine and Health Management, Huazhong University of Science and Technology, Wuhan, China

**Keywords:** Health workforce, US Department of Health and Human Services, Gender pay gap, Occupational segregation

## Abstract

**Background:**

The gender pay gap in the United States (US) has narrowed over the last several decades, with the female/male earnings ratio in the US increased from about 60% before the 1980s to about 79% by 2014. However, the gender pay gap among the healthcare workforce persists. The objective of this study is to estimate the gender pay gap in the US federal governmental public health workforce during 2010–2018.

**Methods:**

We used an administrative dataset including annual pay rates and job characteristics of employees of the US Department of Health and Human Services. Employees’ gender was classified based on first names. Regression analyses were used to estimate the gender pay gap using the predicted gender.

**Results:**

Female employees of the DHHS earned about 13% less than men in 2010, and 9.2% less in 2018. Occupation, pay plan, and location explained more than half of the gender pay gap. Controlling for job grade further reduces the gap. The unexplained portion of the gender pay gap in 2018 was between 1.0 and 3.5%. Female employees had a slight advantage in terms of pay increase over the study period.

**Conclusions:**

While the gender pay gap has narrowed within the last two decades, the pay gap between female and male employees in the federal governmental public health workforce persists and warrants continuing attention and research. Continued efforts should be implemented to reduce the gender pay gap among the health workforce.

## Background

The United States (US) has seen a narrowing gender pay gap over the last two decades [1]. Although further efforts to address gender-based inequities in pay have been stalled, Congress restored the requirement for equal pay for equal work by passing the Lily Ledbetter Fair Pay Act in 2009 [2]. The female/male earnings ratio in the US has increased from about 60% before the 1980s to about 79% by 2014 [1]. The gender pay gap among the healthcare workforce continues to exist and is considered a social determinant of health [3]. For instance, male nurses earned 27.9% more than female nurses in 2013 in the US although the gap reduced to about 9.8% once work hours were controlled [4].

To put it into context, we compare the gender pay gap in the US with other countries. In the UK, female chief executive officers (CEOs) had increased their representation from 37 to 47% during 2012–2018, with a gender pay gap narrowed to become insignificant [5]. However, a recent review reported a gender pay gap in England is 24.4% for hospital doctors, 33.5% for general practitioners, and 21.4% for clinical academics [6]. A gender-imbalanced distribution exists among the hierarchical positions at the Mexican National Institutes of Health, with the gender pay gap mainly at the highest and the lowest ranks [7]. Gender wage gap varied across occupations among a sample of 10,066 Australian full-time employees within the health sector during 2005–2006, with an adjusted pay gap of 16.7% [8].

Examining the gender pay gap in the public sector is of particular interest because public entities often have a mandate of equal pay for equal work. In the US, the Equal Pay Act of 1963 requires equal pay between female and male employees in the same establishment for equal work, defined in terms of requirements of skill, effort, and responsibility. The Lily Ledbetter Fair Pay Act of 2009 removed the restriction on the time period for filing complaints of employment discrimination in pay. However, efforts to increase pay transparency and strengthen federal enforcement tools have been stalled in Congress [9]. While narrowing, the gender pay gap persists among public employees at various levels. Chen and Crown found a gender pay gap of 11% among regular, tenure-track faculty in a public university in 2016, i.e., female employees earn 89 cents for every dollar paid to male employees [10]. Another study finds that males earned 29.6% more than females among US primary and secondary school teachers, although the adjusted gap reduced to 14.6% [4]. The US federal government, the largest public employer in the country, has seen the gender pay gap among its workforce declined from 28% in 1988 to 19% in 1998, and to 11% in 2008 [11]. An agency-specific study produces similar findings, with a narrowed gap from 22–31% in 1994 to 10–27% in 2008. [12] The US Government Accountability Office (GAO) reported an 11 cents difference in pay between male and female federal employees in 2007, among which 7 cents cannot be explained with occupation or education [13]. As the acquisition of personnel data for research purposes often involve a time lag, earlier studies, including a paper published in 2019, have only been able to examine the federal workforce during 1988–2008 [11, 12, 14].

Several strands of literature exist in explaining the gender pay gap. The economic literature decomposes the gender pay gap into contributions of human capital, occupational segregation, and an unexplained portion that is often considered evidence of discrimination [12–14]. Other studies have hypothesized that work hours and wage-setting institutions contributed to the gender pay gap [15–17]. Psychological attitudes towards competition might have accounted for some but a limited portion of the gender pay gap [18]. Childbearing and family roles may have also contributed to the gender difference in career advancement and thus pay [19]. However, the GAO study suggested that unpaid leave and break in service could explain less than one percent of the gender pay gap [13].

The US Department of Health and Human Services (DHHS) is a cabinet-level agency of the federal government with more than 80,000 employees [20]. While DHHS workforce is only a small fraction of the roughly 20 million of healthcare workforce in the nation, it comprises over a quarter of the US governmental public health workforce [21, 22]. Most of the DHHS employees are skilled workers with special knowledge on health services, medicine, and other allied health or supporting disciplines, representing a significant portion of the public health workforce. In addition, the DHHS is considered as a gender-neutral agency versus masculine agencies such as the Department of Homeland Security [12]. Earlier studies have taken an agency-specific approach to separate the impact of organizational cultures and social norms on the gender pay gap [12, 14]. However, none of the prior studies has focused on the federal governmental health workforce, and there is a lack of updated analyses on the gender pay gap since 2008.

This paper intends to examine and explain the gender pay gap among the employees of the US DHHS during 2010–2018. A key innovation is our use of an online federal employee salary database and the recently released Social Security Administration (SSA) database of first names of newborns. The online portal obtained the salary information of federal employees through Freedom of Information Act (FOIA) requests [23]. We use the SSA online database, supplemented with an online state employee database to predict the gender information. Our results provide a timely update on the gender pay gap within the US federal governmental health workforce.

## Method

### Data

The primary data of this study were extracted from FedsDataCenter.com using Python [23]. FedSmith Inc. acquired the salary data of federal employees through FOIA requests and provided the information for free through FedsDataCenter.com. We extracted information on the name and pay of salaried employees of the US DHHS during 2010–2018 except for 2014. The data during 2010–2013 were scrapped for an earlier project. The data for 2014 were not available when we started extracting data again in 2019. Besides pay and full name, data fields included the location where the employee is stationed, job title, pay plan, and pay grade. All data are publicly available.

The key dependent variable was the pre-tax salary, the annual pay rate documented in the employee's human resource record. The pay rate might differ from the employee's exact pay during the year if the employee had any personnel changes in the system. However, it reflected employees’ base pay rate during the fiscal year.

Location was an important factor in determining federal employees' salaries. As a measure to adjust for the cost of living, federal salaries varied across locations (domestic and foreign) with a multiplicative factor, which was authorized under Title 5 US Code Sect. 5304 and applicable to most employees of GS and several other pay plans [24]. Pay plans were a set of tables or arrays of pay rates "prescribed by law or other authoritative source that establishes the basic pay rates for certain employees" [25]. The most common federal pay plan was the GS plan.

GS grade (or pay grade) is the 15 levels on the bureaucratic ladder under the GS plan, ranging from GS-1 to GS-15 [24]. Higher pay grades often require advanced degrees and thus higher pay rates. GS-14 and GS-15 typically involve supervisory responsibilities. For each grade level, there were ten "steps" through which the employees may advance by accumulating sufficient length of service or by merit.

We proxy occupation using job titles, descriptive texts provided by agency human resource departments. However, the accuracy of the texts relies on the practice of the specific human resource specialist. Multiple variations or abbreviations of a job title might exist. These issues could lead to over-stratified categories but would not affect the main results of our analysis.

Names of the DHHS employees were also available as part of the FOIA requests [26].

### Predicting gender based on first names

Predicting gender base on first names had been increasingly applied in research using names extracted online [27, 28]. To determine the gender of the employees, we used the SSA database of baby names with at least five uses in a year from 1940 to 2000. We ascertained an HHS employee's gender by assigning the gender that appeared in the database at least 95% of the time.

The SSA database contained the first names of those who were born in the US thus might not capture the first names of foreign-born employees. To augment the SSA database, we used a database from the state of Iowa [26], which publishes salary information of state employees, in addition to their full name and gender. The Iowa database helped us to determine the gender of some employees whose first names had not appeared in the SSA records, including hyphened compound first names and first names that came from foreign languages.

By using the SSA records and the Iowa database, we were able to assign genders to 6,890 female first names and 3,542 male first names, for 111,320 unique names (first name and family name) and 518,575 records over the 8 years.

### Analytic approaches

Following prior studies, we regressed the logarithm of the annual pay on a set of control variables and the assigned gender indicator for each yearly cross-section [10, 29]. The first set of control variables included the fixed effects of location. The logarithm of salary allowed the multiplicative nature of the locality adjustment. The second set included fixed effects of job title and pay plan to control for the effects of occupation and managerial responsibilities. The third set of control variables added the fixed effects of job grade, which approximates the impact of promotion and managerial responsibilities.

Utilizing the longitudinal nature of the data, we examined the gender difference in salary increase over the study period. However, the DHHS employee records had multiple duplicate names in a year. Because we could not differentiate duplicate names over time, we removed any names that appeared more than once in a given year. We constructed a panel over time but opted not to use the employee fixed effects because the fixed effects will absorb the gender difference. The large number of fixed effects also exceeds our available computation capacity. With the remaining sample of unique names, we subtracted the logarithm of pay for an employee in the previous year from that in the current year and used the difference as a dependent variable. We regressed the resulted difference over the three sets of control variables.

### Ethics review

Our research is a secondary analysis based on public records. The University of Georgia Institutional Review Board determined the research does not involve human subjects on March 9, 2021 (IRB ID: PROJECT00003778).

## Results

We have identified the gender of most employees during 2010–2018 (Table 1), resulting from a low of 51,943 in 2015 to a high of 74,511 employees in 2012 used in the analysis. A sharp drop in the number of DHHS employees shown in the FedsDataCenter.com data occurred in 2015.

**Table 1:** Identifying the gender of DHHS employees based on first names

Year	Total number of DHHS employees	% of DHHS employees whose gender is not identified	Among those whose gender is identified		Average pay		Female/male pay ratio (%)
			% of GS employees	% Female	Male	Female	
2010	81,111	11.5%	82.2	58.9	$94,675	$82,370	87.0
2011	83,277	11.8%	82.1	58.8	$94,845	$83,653	88.2
2012	84,636	12.0%	82.2	58.8	$95,480	$84,693	88.7
2013	84,595	12.2%	82.9	58.8	$96,063	$86,090	89.6
2015	60,243	13.8%	82.7	59.2	$100,941	$90,592	89.7
2016	61,399	14.6%	83.8	59.0	$101,695	$91,689	90.2
2017	70,831	15.0%	83.7	59.4	$105,582	$95,766	90.7
2018	73,858	15.4%	83.	59.6	$109,542	$99,515	90.8

*DHHS* US Department of Health and Human Services, *GS* General Schedule. The number of employees is calculated based on data reported by the FedsDataCenter.com

GS employees comprise about 82–83% of the DHHS employee. The percent of female employees among DHHS employees has been stable at around 59% (Table 1), while the percentage is higher at roughly 63% among GS employees.

Comparing the average pay reveals a narrowing but persistent gender pay gap, starting at about $12,305 (13%) in 2010 to roughly $10,027 (9.2%) in 2018 (calculations based on Table 1). Table 2 provides, for each GS grade, the percentage of female employees, mean pay rates of female and male employees, and the female/male pay ratio. The percentage of female employees for GS-15 is the lowest. The grade-specific female/male pay ratios are close to one but with the highest ratio at 1.08 for GS-7 and then decline in general (with the exception of GS-10) as the grade progresses. Controlling for location has a marginal reduction of the gap in terms of the percentage difference (Table 3). For example, the regression produces a negative coefficient of − 0.131 for being female in 2010, suggesting females earn 13.1% less than males in 2010. Similarly, females earn 8.3% less than males in 2018.

**Table 2:** Percentage of female employees among GS DHHS employees and salary comparison between male and female employees by grade

Grade	Male	Female	%Female	Female/male pay ratio
1	$22,474	$22,423	73.8	1.00
2	$25,570	$25,138	51.2	0.98
3	$27,942	$27,756	52.4	0.99
4	$31,971	$32,334	72.0	1.01
5	$35,209	$36,934	71.7	1.05
6	$40,965	$42,353	72.0	1.03
7	$43,261	$46,533	56.8	1.08
8	$52,688	$54,946	66.9	1.04
9	$55,159	$56,899	69.4	1.03
10	$68,784	$69,472	54.3	1.01
11	$67,707	$69,727	64.4	1.03
12	$82,416	$85,105	58.7	1.03
13	$103,045	$103,564	60.7	1.01
14	$124,413	$124,717	56.1	1.00
15	$148,933	$148,770	50.5	1.00

*DHHS* US Department of Health and Human Services, *GS* General Schedule

**Table 3:** Gender pay gap by year among US DHHS employees during 2010–2018

Dep. Var: ln(salary)	2010	2011	2012	2013	2015	2016	2017	2018
Panel 1: Controlling location								
Female	− 0.131*	− 0.118*	− 0.111*	− 0.101*	− 0.094*	− 0.086*	− 0.084*	− 0.083*
	(0.003)	(0.003)	(0.003)	(0.003)	(0.004)	(0.004)	(0.003)	(0.003)
*R*^*2*^	0.323	0.336	0.338	0.348	0.346	0.351	0.343	0.346
Panel 2: Controlling location, occupation, and pay plan								
Female	− 0.053*	− 0.049*	− 0.050*	− 0.046*	− 0.044*	− 0.035*	− 0.028*	− 0.035*
	(0.002)	(0.002)	(0.002)	(0.002)	(0.003)	(0.002)	(0.003)	(0.002)
*R*^*2*^	0.736	0.734	0.740	0.739	0.722	0.724	0.664	0.736
Panel 3: Controlling location, occupation, pay plan, and grade								
Female	− 0.018*	− 0.019*	− 0.020*	− 0.017*	− 0.016*	− 0.010*	− 0.008*	− 0.010*
	(0.001)	(0.001)	(0.001)	(0.001)	(0.001)	(0.001)	(0.002)	(0.001)
*R*^*2*^	0.897	0.889	0.887	0.892	0.886	0.907	0.862	0.903

*DHHS* US Department of Health and Human Services; standard errors in parentheses; * p < 0.0001

After controlling the fixed effects of job title and pay plan in addition to location, the gender pay gap narrowed substantially. The remaining unexplained gender pay gap is 5.3% in 2010 and 3.5% in 2018, suggesting that females earn 5.3% less than males in 2010 and 3.5% less in 2018, accounting for the location factors, occupation selection (or occupational segregation), and selection into administrative duties. The last set of control variables added an additional set of the fixed effects of grade, resulting in the smallest unexplained gap of 1.8% in 2010 and 1.0% in 2018 (Table 3).

The regression on the rate of pay increase suggested female employees do have a slightly higher rate (0.82 percentage point) of pay increases than male employees controlling only location (Table 4). However, once occupation and pay plans are controlled, the advantage narrowed down to 0.39 percentage points. If the grade level is also controlled, the difference is even smaller—suggesting that female employees have a rate of pay increase that is 0.24 percentage point higher than males. Similar results are observed for the sample of GS employees.

**Table 4:** Gender gap in pay growth among US DHHS employees during 2010–2018

Dep var: ln(salary_t_/salary_t-1_)	(1)	(2)	(3)
All HHS employees who do not have duplicate names in the year			
Female	0.0082*	0.0039*	0.0024*
	(0.0006)	(0.0006)	(0.0006)
*R*^*2*^	0.059	0.094	0.100
HHS GS employees who do not have duplicate names in the year			
Female	0.0064*	0.0027*	0.0010
	(0.0006)	(0.0006)	(0.0006)
*R*^*2*^	0.072	0.108	0.117

*DHHS* US Department of Health and Human Services; Standard errors in parentheses; * p < 0.0001; Model 1 controls for the fixed effects of location; Model 2 controls for the fixed effects of location, job title, and pay plan; Model 3 controls for the fixed effects of location, job title, pay plan, and grade

## Discussion

**Fig. 1:**
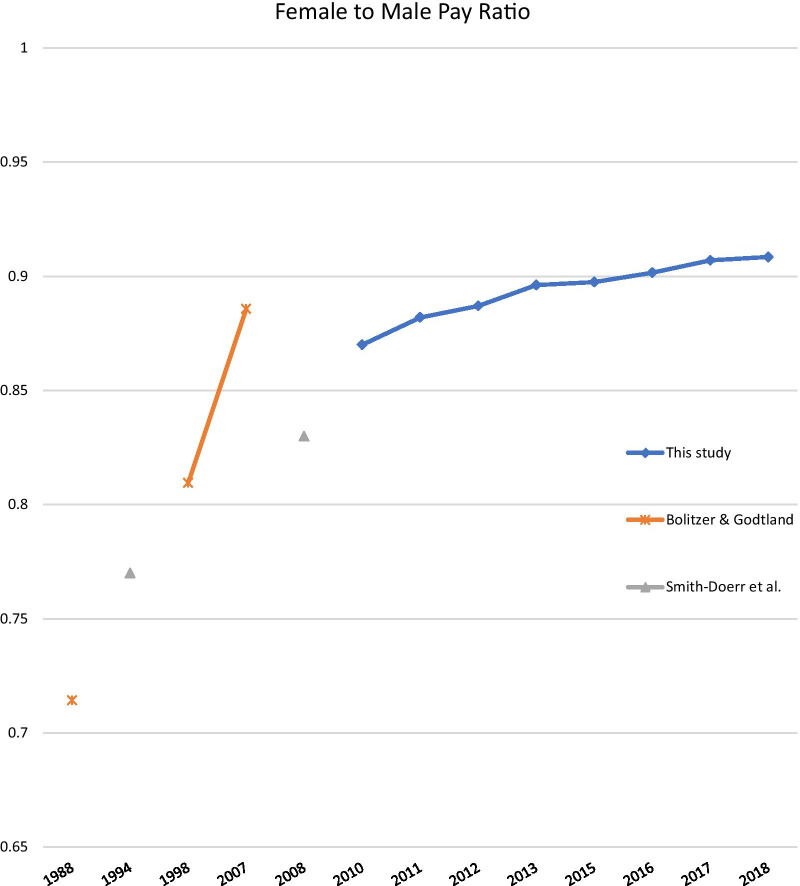
Estimated female-to-male pay ratio among the US Federal workforce

This study use an administrative dataset of annual pay rates of US DHHS employees, coupled with use of the SSA first name database and a database of the State of Iowa employee salaries, to assess the gender pay gap among the federal governmental public health workforce. We find a narrowing but persisting gender pay gap, after controlling for location, job title, pay plan, and job grade. Compared to the raw and adjusted pay gap of 11% and 4% in 2007 identified by the GAO for the federal workforce, the gender pay gap among DHHS employees has narrowed significantly, with raw and adjusted pay gaps at 8.3% and 1%. The estimated trend of a narrowing gender pay gap observed in this study is consistent with earlier research examining different components of the federal workforce [11–13] and the trend across the high-income countries [5]. Even though this study and two earlier publications have examined different parts of the federal government, the results on the female-to-male pay ratio over time reveal a consistent trend of a narrowing gender pay gap (Fig. 1).

Our analysis of the data from FedsDataCenter.com provides a close approximation of the DHHS administrative record. Our estimated proportion of females in the DHHS workforce is slightly less than 60%, which is consistent with the proportion reported in earlier studies using administrative data in 2006 [19] or employee survey data in 2004 [30]. This may indicate a willingness of female workers to self-select into health-related fields [7]. The estimated gender pay gap (a female-to-male pay ratio of 91.8% in 2018) is similar to what is published on Fedscope.com, which shows an average pay of $98,409 for female and $106,723 for male among DHHS employees in 2018, resulting in a female-to-male pay ratio of 92% [31].

The narrowing gender pay gap can be explained by the increase in education and experience as well as an increased entry of females into occupations that had been dominated by males [1, 11–13, 19, 32]. The GAO reported has identified a contributing factor that may be both encouraging and concerning—the clerical positions that were dominated by female employees have been diminishing in the federal government [13]. The diminishing clerical positions is encouraging because it may indicate that technological and social changes have enabled and empowered females to enter a broad array of fields. However, the federal government's use of contractors may lead to more females being hired as contractors, whose pay will not appear in the database thus unaccounted for in the calculation of gender pay gaps [33].

Grade, or the position in the bureaucratic hierarchy, is an essential element of pay for federal employees. The GAO report, though acknowledged the narrowing gender pay gap among the federal workforce, has also pointed to an important concern—the portion of the gender pay gap that cannot be explained by occupation, experience, and education increased from 2 cents in 1988 to 7 cents in 2007. Our study suggests that occupation and pay plan explained about 8 cents (the difference between − 0.053 and − 0.131) of the gender pay gap in 2010, and 5 cents in 2018. Including the pay grade explains an additional 2.5 cents in 2010 and 2018. However, because pay grade is a proxy of promotion, it may have components that can be explained by education, years of government service, as well as an unexplained portion. Similar to what has been observed among the employees of the Mexican National Institutes of Health, [7] the distribution of female employees across different grades of the GS has been uneven, with the smallest portion of female employees reaching the highest grade, GS-15, and higher percentages of female employees at lower grade levels (e.g., GS-13). Given a grade level, females earn slightly more than males at lower grade levels, but the advantage starts to disappear at grade level 13 (Table 2). This, coupled with the higher female average pay, may indicate that female employees tend to stay longer on the higher end of the 10-step band of lower grade levels, being passed on promotion opportunities. To summarize, the unexplained portion of the gender pay gap may lie within the interval determined by models 2 and 3, i.e., between 1.8 and 5.3 cents in 2010 and between 1.0 and 3.5 cents in 2018.

Competing theories exist in explaining the unexplained portion of the gender pay gap, e.g., childcare and wage structure [1, 15, 16], gender difference in psychological attitude [18], unpaid overtime [8], and the lack of role models [19]. Employee viewpoint survey or focus group interviews may help to pinpoint the exact causes of the remaining gender pay gaps.

Policies and interventions to reduce the gender pay gap include a one-time pay raise for female employees as the University of Essex did [34], and the Denmark and UK mandates for institutions to publish gender pay gaps. Such mandates have been shown to have narrowed the gender pay gap in the UK and Denmark [35–37]. Our results provide support for the proposed Pay Check Fairness Act (H.R.7, 117th US Congress) to improve transparency, to protect female employees’ right to challenge pay discrimination, and to hold employers accountable.

Our study does send a message to the international community: a high-income, well-developed civil society may not be immune from gender pay disparities. Continued efforts to eliminate gender disparities in pay, promotion, and leadership roles are needed anywhere and everywhere.

### Limitations

This study has several limitations. First, FOIA requests are handled within a set timeframe, often at the price of quality control. Agency might also have different policies as to what information to be withheld over time. We have seen the variation in our sample size, which dipped after 2014. The direction of the impact of such variation on our estimated gender pay gap is unclear. However, if the quality control or information withheld does not vary systematically across the gender groups, our results will not be critically impacted, which seems to be the case as we have seen the consistent trend in the estimated gender pay gap. Second, we have more than 10% of the first names that cannot be assigned a gender. Some of the names are gender-neutral names, such as "Drew" or "Robin", that neither gender had been used at least 95% of the time during 1940–2000. Though individuals with those names are slightly more likely to be female, as shown in the SSA data, thus our estimated gender pay gap may bias upward as those who cannot be assigned a gender have a higher average salary. Some of the other names that cannot be assigned gender are from foreign languages, which is difficult to determine the direction of the biases from dropping those names. However, the resulted bias may be negligible because the Fedscopes.com data provide strong support for our results. Third, the job titles provided through FOIA requests are often unstructured texts with many different forms of abbreviations and variations. This leads to issues where multiple fixed effects are estimated for the same occupation. However, the additional degree of freedom is trivial and should not impact our point estimates, given the large sample size of this study. Fourth, we do not have information on experience, education, and race and ethnicity. Meanwhile, pay grade may capture some of the differences in experience and education because specific requirements on education and experience exist for progressing to a particular pay grade. However, we acknowledge that our estimates may not reveal the important pattern of gender pay gaps by race and ethnicity due to the data limitation.

## Conclusions

The gender pay gap is a social determinant of health that affects the outcomes of the public health system. A narrowing gender pay gap, from 13% in 2010 to 9.2% in 2018 at DHHS, the nation's premier health agency, is encouraging news for practitioners. Identifying the contributors to the gap will pave the way to reducing and ultimately eliminating the gender pay gap.

In line with the mandate to publish gender pay gaps in the UK, our research calls for added data transparency. While the federal initiative to publish data through data.gov is commendable, making the data available to the research community at the same speed as responding to the FOIA requests would improve the efficiency of research and evaluation of gender pay equity among the federal workforce. Renewed efforts to reintroduce the Paycheck Fairness Act is an important step for the American public to close the gender pay gap.

## Data Availability

The datasets used and/or analysed during the current study are available from the corresponding author on reasonable request.
